# Home: The place the older adult can not imagine living without

**DOI:** 10.1186/1471-2318-11-10

**Published:** 2011-03-17

**Authors:** Catharina Gillsjö, Donna Schwartz-Barcott, Iréne von Post

**Affiliations:** 1 School of Life Sciences, University of Skövde, Sweden; 2 College of Nursing, University of Rhode Island, USA; 3 Department of Caring Science, Åbo Academy University, Vasa, Finland

## Abstract

**Background:**

Rapidly aging populations with an increased desire to remain at home and changes in health policy that promote the transfer of health care from formal places, as hospitals and institutions, to the more informal setting of one's home support the need for further research that is designed specifically to understand the experience of home among older adults. Yet, little is known among health care providers about the older adult's experience of home. The aim of this study was to understand the experience of home as experienced by older adults living in a rural community in Sweden.

**Methods:**

Hermeneutical interpretation, as developed by von Post and Eriksson and based on Gadamer's philosophical hermeneutics, was used to interpret interviews with six older adults. The interpretation included a self examination of the researcher's experiences and prejudices and proceeded through several readings which integrated the text with the reader, allowed new questions to emerge, fused the horizons, summarized main and sub-themes and allowed a new understanding to emerge.

**Results:**

Two main and six sub-themes emerged. Home was experienced as the place the older adult could not imagine living without but also as the place one might be forced to leave. The older adult's thoughts vacillated between the well known present and all its comforts and the unknown future with all its questions and fears, including the underlying threat of loosing one's home.

**Conclusions:**

Home has become so integral to life itself and such an intimate part of the older adult's being that when older adults lose their home, they also loose the place closest to their heart, the place where they are at home and can maintain their identity, integrity and way of living. Additional effort needs to be made to understand the older adult's experience of home within home health care in order to minimize intrusion and maximize care. There is a need to more fully explore the older adult's experience with health care providers in the home and its impact on the older adult's sense of "being at home" and their health and overall well-being.

## Background

An 89 year old woman was offered an apartment in a residential facility for older adults. She had four days to decide whether or not to leave the house that had been her home for 79 years, almost her entire lifetime. It was a very hard decision. It was difficult to "leave everything", including her beloved garden. She had second thoughts regarding the decision she had made and kept thinking that maybe she could have stayed in her home longer with the help of home health care providers? She could not stop thinking about her home and worried about the future [1]. What is it about a place that the older adult calls and experiences as home that could cause such distress?

Home is a value laden [2] everyday term [3-6] that easily can be taken for granted [7] even though it often is the most central place in one's life [8-12]. Numerous authors have noted that for older adults, home is especially important and central to daily life as it becomes a major base of activity as many grow old [13-15]. The passing of time and past memories embedded in the home tend to deepen the older adult's sense of rootedness and the desire to stay and grow old in one's present home often becomes strong [15-17].

Over the last 30 years, there has been increased interest among scholars about the notion of home and a distinctive body of scholarly literature around the topic has emerged. The focus on home as an important area for scientific inquiry began to appear in the mid 1970s as did a seminal article on the concept of home by an architect named Hayward [3]. Between 1989 and 1992 a series of international conferences took place in Sweden, New York, Norway and Denmark and included scholars from diverse disciplines ranging from architecture and geography to social sciences and philosophy [18]. The dialogue about the topic continued in a book edited by Rowles and Chaudhury [19] on the home and aging. Issues of concept clarification have continued to be central in these discussions. However, less progress has been made in defining the concept, than in identifying the personal meanings of home and how these have been influenced by historical, social, cultural factors as well as demographic characteristics [18].

In nursing, home is a well established arena for nurses working in home health care. In fact, in early Roman writings the home appeared as a setting where health promotion and care were given by nurses [20]. In the late 1800s, it became linked with organized community health care nursing and the initiation of home visiting [20-22]. Byrd [22] investigated the concept of home visiting since it has become a central nursing intervention within home health care. Over the last 30 years, home as an arena where health care is given and received has increased enormously due to a rapidly aging population and a desire to remain at home [9,23] as well as change in health policy with a transfer of care from formal places such as hospitals and institutions to more informal settings such as home [11,12,24-26].

The need to look more closely at home has also been raised cogently by Williams and a group of colleagues in medical geography. These authors have begun to conceptualize the home as a therapeutic environment and to examine the impact, both positive and negative, of home care services on the relationship between the client and the home environment. They acknowledged the possibility of intruding and violating the client in the home [11]. Also, within occupational therapy there has been increasing recognition of the home as a central place in the lives of older adults and the potential disruptive impact of health care providers in this setting [15]. As Hawkins and Stewart [24] point out, there tends to be an assumption that the home is a neutral territory where new relationships, occupational roles and functions are expected to be incorporated without problems. Yet, Steward [27] has argued that the mere presence of health care providers in the home changes it from a private space to a public arena and may in turn disrupt the boundaries and the older adults sense of home. Additionally, research by Roush and Cox [28] and Swenson [10] showed that older adults experiences of home health care have an impact on their notion of health and well-being.

However in spite of the increased interest and voluminous literature, Gillsjö and Schwartz-Barcott [1] found in a recent and extensive cross-disciplinary review that there is no single comprehensive and measurable definition of the concept of home. Home instead was identified as *a place, a relationship *and *an experience*. The authors found that by far the most fundamental use of the term was in reference to a place. However, almost immediately any discussion of the concept also addressed it as a special relationship between a place and the individual who called it a home. The phrase "feeling at home" often was used to convey this emotionally based relationship that developed overtime. Additionally a number of authors looked at home as an experience, focusing on "being at home". Gillsjö and Schwartz-Barcott [1] concluded that home as a scientific concept could be defined as a place to which one is attached, feels comfortable and secure and has the experience of dwelling. This definition allows one to distinguish a home from a house (a residential structure) and from personal meanings (whether positive of negative) of home, two major points of confusion found in the literature [18].

The least empirically grounded of these components was definitely the experience of home. Dovey [29-31] highlighted the experience as "being at home" and saw it as an insider's experience and as always unique [30]. According to Dovey [30], it is a mode of being, where one is are 'oriented within a spacial, temporal and sociocultural order' (p.35) that one understands. However, Dovey's writings on the experience of home were based on philosophical analysis rather than on empirical research. This lack of empirical research also was noted by Moore [32] who argued for a critical examination of this perspective, one that could be informed by a more direct link with empirical inquiry. Additionally, Gurney and Means [33] highlighted the limited literature on the subject among older adults and argued the need for empirical research on the experience of home among older adults.

There has been some empirical research in nursing where the experience of home has been partially illuminated [4,10,34,35]. Furthermore, the importance of the lived experience of "being at home" has drawn attention as researchers have looked more closely at the experiences of older adults who have moved into assisted living facilities. Hammer [36] focused on older adults' experiences of being at home among those who had been forced to leave their homes and move to facilities that provided services for older adults. Rapidly aging populations with an increased desire to remain at home [9,23] and changes in health policy that promote the transfer of health care from formal places (e.g. hospitals and institutions) to the more informal setting of one's home [11,12,24-26] support the need for further research that is designed specifically to understand the experience of home among older adults.

The aim of this study was to use individual interviews and hermeneutical text interpretation to gain an understanding of the experience of home among older adults. The interviewees were community dwelling older adults living in a rural community in the west region of Sweden.

## Methods

In this study six older adults were interviewed and von Post and Eriksson's [37] approach to hermeneutical interpretation of a text was used to gain a deeper understanding, rather than simply a description of the older adult's experience of home. This approach was drawn from Gadamer's hermeneutical philosophy [38].

Qualitative research as a serious mode of scientific inquiry in the area of health care emerged in 1980s. Findings from qualitative research have contributed to an increase in practical knowledge in health care and have been incorporated in standard hierarchies of evidence used for establishing evidence-based practices [39,40].

The qualitative interview is theme oriented in which an interaction takes place in the conversation between the interviewee and the researcher about a specific subject. The interviewee gives a description with emphasize on what is considered to be important and the researcher is able to obtain confirmation of interpretations of both verbal and non verbal expressions during the interview. In contrast with the highly structured questionnaire, the qualitative interview is not focused on objective, reproducible and quantified data. However, this type of open extensive interview provides an opportunity to gain a broader and deeper understanding that goes beyond the interviewee's self-understanding of the central theme [41,42].

### Participants

A purposeful sample was drawn of older adults who were living in a small rural community in Sweden and who were able and willing to talk about what a home is to them. It included six older adults, four women and two men ranging in age from 77 to 89 years. The participants included three widows, one married man and one married couple. Two of the women lived alone in their houses and one in an apartment. The married man lived in a house and the married couple in an apartment. Four of the participants were contacted through district nurses, and two were contacted on a private basis. Additionally, four of the participants had daily or weekly service from home health care.

### Data collection

Data were collected by a qualitative research interviewing approach developed by Kvale [41,42] and appropriate for hermeneutical interpretation. Participants were initially contacted by phone by the researcher who described the study. This was followed by an informational letter that included a written description of the study and the informed consent. The participants were interviewed by the first author in their place of residence. All of them were interviewed once except from the married couple for whom a second time was needed to clarify some issues. Each interview was conducted as an open theme oriented conversation around the older adult's experience of home, rather than person oriented. Before the interview even began the participants spontaneously commented on how difficult they thought it would be to talk about their own experience of home since they had not reflected before on their home in this way. However, initially when asked a relatively general and abstract question, *what is a home to you*, the participants were able to begin to talk about their home. The opening question allowed time to develop some level of trust in the conversation and gave the older adults time to think about what they wanted to say about their experience of home. It also allowed the first author time to reflect upon and decide about subsequent questions based upon what had or had not been expressed or needed to be confirmed or clarified to achieve a rich description of the older adult's experience of home. Subsequently, during the interview more specific and concrete questions were asked such as: *Tell me about your home(s)? What makes this a home to you? *As the interviews progressed, participants were asked questions like, *would it be possible to move this home somewhere else and would it still be a home to you*, to help them reflect upon and describe their experience of home. Interviews lasted from 30-65 minutes, were tape-recorded and transcribed verbatim by the first author.

### Ethical consideration

The study was approved by the Ethics Committee, University of Gothenburg in Sweden (Ö 191-03). Additional approval was given by the heads of the community social welfare and health services. Participants received a letter with information about the study. They consented verbally and in writing to participate in the study. Additionally, several reminders were given about the voluntarily nature of their participation prior to the interviews. The identity of participants was protected by confidential handling of data.

### Hermeneutical text interpretation

Von Post and Eriksson's [37] approach to hermeneutical text interpretation was used to interpret the interviews with the six older adults. This approach was chosen because it seeks to understand the meaning of the text more than how it was created. Additionally, the authors emphasise the importance and role of the nurse researcher's professional and personal experiences and prejudices in interpreting the text.

The above approach was based on Gadamer's [38] hermeneutical philosophy, in which language has an important role in creating the existential world in which reality may be revealed and interpreted. Through an open dialog the reader (researcher) seeks to interpret and understand the meaning of the text and not the underlying purpose behind the interviewees' responses. According to Gadamer [38], it is extremely important that the researcher describe the circumstances, most notably one's pre-understandings, under which the interpretation of the text is made. It is this awareness of one's pre-understandings during the interpretation of the text that helps the researcher in recognizing, interpreting and understanding themes that emerge out of the text[43].

Gadamer [38] argues that all people have an existential pre-understanding of life. This is a prerequisite to be able to interpret, understand and acquire knowledge about the reality that the text conveys through language. However, one has to recognize, reflect upon and question one's pre-understanding to gain insight into one's prejudices to increase understanding of self. An open mind (where one's prejudices are used to enhance rather than lead to misunderstanding) is required to be able to see and understand the "otherness" of a phenomenon in the world. The process of understanding includes a dialogue between the text and the reader in which a fusion of horizons of understanding emerges and a new horizon opens up. The dialectic interplay and oscillation between the parts and the whole expresses the process of thinking and interpreting used to expand the horizon of understanding, which is called the hermeneutic circle.

Von Post and Eriksson's [37] approach to interpretation begins with the researcher's self examination of professional and personal experiences and prejudices and proceeds through several readings that focus on integrating the text with the reader, fusing horizons, allowing new questions to emerge, summarizing main and sub-themes and allowing a new understanding to emerge. In these reading one moves back and forth rather than through a specific sequencing of steps. Each point of focus is described below in relation to its use in this study.

According to von Post and Eriksson [37], professional pre-understanding is more than purely existential pre-understanding. Professional pre-understanding is the result of one's professional education and experience as a nurse and as a part of the subculture of nursing. More specifically this pre-understanding is made up of the researcher's perspective on caring, knowledge of medicine, personal values, prejudices and commitments and the ethics that guide nursing as well as one's experience as a nurse. One has to be aware of, take into account and question one's professional pre-understanding in a way that it neither enhances nor clouds one's view.

The first author's pre-understandings arose from personal experience of being at home in more than one place. Additionally, the first author has worked with older adults in a nursing-home setting and in community based home health care. Prior to the first reading, the first author reflected on her past professional and existential pre-understandings.

#### Integrating the text with the reader

The interviews were completed and compiled as the text. The first reading began as an open reading moving from the beginning to the end of the text in order to become familiar with the text and integrate it with the reader. The text spoke to the first author and her pre-understandings (existential and professional role as a district nurse) made the text understandable. While reading, questions began to emerge such as *Is this the home, do I really understand what it is like? *The reader listened and allowed the text to say something true about reality, here being the home, without questioning the objective nature of that reality [37].

#### Fusing of horizons

The text was carefully reread with an open mind and a dialogue was begun in which the horizon of the text and that of the reader were brought into a relationship with each other. The reality of the text became a part of the reader. The existential and professional pre-understanding had to be brought into awareness and taken into account again in relation to the reading of the text [37]. In the fusion of horizons it became obvious that the home was very important and close to one's heart. The home was seen both as something concrete and as something abstract. At this point the initial interview question re-emerged but with more urgency: w*hat really is a home to an older adult?*

#### Allowing new questions to emerge

As the dialogue continued with the text, the home emerged as an experience, not as an entity, and the new question then was: *how does the adult experience their home? *This is similar to the original aim of this study but it was now more focused. It did not reflect the breath of the term home as originally conceived. Additionally, it encompassed more than the original meaning of the word experience as simply "being at home". Subsequently the text was carefully reread again in order to discover answers to this new question. Significant expressions, quotations with common and distinguishing qualities were drawn from the text [37].

#### Summarizing main- and sub-themes

The text with its significant expressions and quotations was carefully read through, looking for its meaning; the real common quality of all significant expression and quotations. This common quality was formed in two main themes and then distinctive qualities were looked for and resulted in six sub themes [37].

#### Allowing a new understanding to emerge

**Figure 1:**
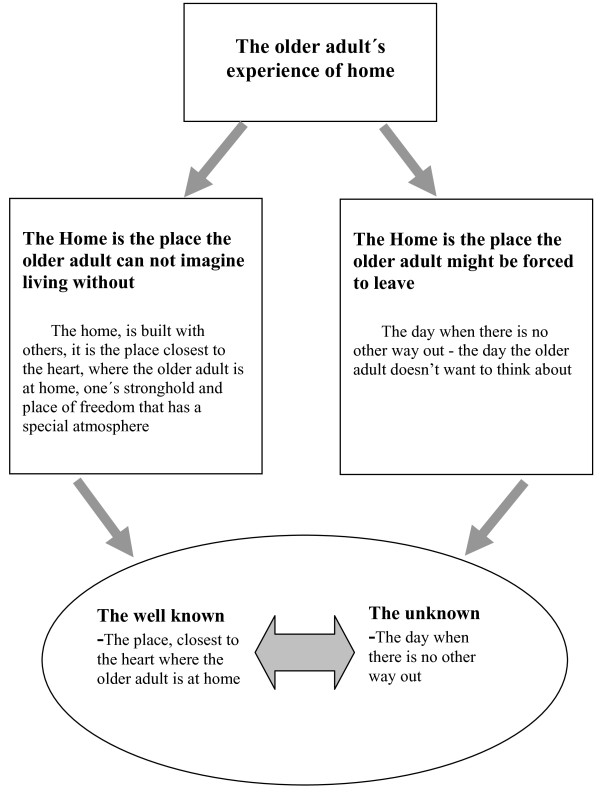
The older adult's experience of home: A vacillating movement between the well known present and the unknown future.

The whole text was read once again to reconfirm all themes against the text as a whole in a search for a new understanding of the whole, from its parts and the parts from the whole. This process of understanding involved an abstraction of the main themes and the sub-themes to form a new understanding, a coherent whole (portrayed later in Figure 1), which was seen as valid and free from inner contradictions [37,41].

## Results

The first main-theme is that home is *the place the older adult can not imagine living without*. The following four sub-themes support this theme: *The home is built with others. The home is the place closest to the heart, where the older adult is at home. The home is one's stronghold and place of freedom. The home has a special atmosphere*. The second main-theme is *the home, a place the older adult might be forced to leave *with the sub-themes: *The day when there is no other way out *and *The day the older adult doesn't want to think about*. The themes are presented below and illustrated by quotes from throughout the text.

### The home, the place the older adult can not imagine living without

The home was not simply a place nor a discrete entity but something that had been built over time with others and emanated a special atmosphere. It had become integral to daily life and to one's sense of self as well as ever so close to one's heart. The older adult could not really imagine its absence.

#### The home is built with others

The home is where the young couple started their common life and built the home together. With joy they built the home up from its foundation. They worked together to make it nice and comfortable. The home grew and little by little it became more and more a home.

"That is the thing one has, well, put together through the years and it becomes a home."

The things the older adult has in the home belong to the home and one wants to be surrounded by them; they make the place a home. These include gifts from their spouse, children, parents and friends or things one has collected or inherited over the years. These things are important to the older adult. A home without decoration and furniture would be almost like a prison.

"Then one has certain things that make a home, inherited things for instance...photos is not the least I think of."

"A proper interior decoration, I think that means a lot. Because a home without furniture becomes a real prison, so to say."

Thus, for the older adult the home was built together with others from its foundation over a long time. In the home, one keeps and protects the things one wants to be surrounded by, things that are important for one's well-being. One imagines that life without these things must be like living in a prison.

#### The home, the place closest to the heart, where the older adult is at home

For the older adult, home is the place where one lives and it is solid ground, where one wants to be and go to, as well as, a place where one is at home. The home belongs to the people living there, it is the place closest to the heart, and a place one can not live without.

"A home is always a home and it is what is closest to my heart."

The home is a place where the older adult lives and where one is deeply rooted. It is a safe place to live everyday life. The older adult wants life to continue in the old tracks and ruts with one's own ongoing habits and routines. The home is something one does not want to let go of because it makes the older adult feel at home.

"I have lived here for many years. It is 54 years this Christmas. And maybe it becomes a matter of habit...one gets so habituated to it."

The home is a place in which the older adult draws strength. It is a place to rest, to relax and find peace and quiet. Family members draw strength from each other. They bond naturally through difficult and happy times and over time become closely united. The home is a place where the children have grown up and a place to which the children can return. The home is a place where it feels comfortable to be alone in or with family and one can be oneself.

"The home is more of the place where one rests...The home is somewhere where one can be oneself."

Thus, the home is the place closest to the older adult's heart where one has lived for a long time and where one's own habits and routines are continuously ongoing, all of which contributes to the feeling of being at home. The home is a source of energy which enables one to live daily life with others or alone. It is the place where the older adult feels comfortable being oneself, which in turn helps to preserve and maintain one's dignity.

#### The home, one's stronghold and place of freedom

For the older adult home is one's own and one's stronghold. It is the place where one is safe and free to choose whom to welcome into one's home. It is the place where one has the power to separate and protect oneself from the outside world. One can choose not to let other people in if one wants to be alone. As a stronghold it is the place where one has the right to decide for oneself and home becomes the safe place in life.

"My home is my stronghold."

"For the home to be my stronghold I have to have a key and be able to lock...The place where I can lock myself in, it is mine."

One's stronghold is a place where the older adult is independent. It becomes a personal place where one has the freedom to do what one wants and has the strength to leave the rest behind. One can be left alone to oneself in the home and does not have to bother others. The freedom to make choices and decisions is important to the older adult.

"I do exactly what I choose...Whatever one says, the freedom is very important."

Thus, the home as a stronghold is a shelter for the older adult's inner self where one's very being and one's free will is protected. The home, is one's stronghold and place of freedom where no outsider can intrude without permission, an inviolable place that in the concrete and in the abstract is inhabited by the human being. The home, the older adult's stronghold is a protective place where one can withdraw to rest, a place that offers piece, quiet and privacy.

#### The home has a special atmosphere

For the older adult home, a special atmosphere pervades in the home. This atmosphere reflects the older adult's values, beliefs, personality and way of life. The atmosphere that pervades in the home gives the older adult a special sense that 'now I am at home,' the moment one steps over the threshold.

"The home is all this...belongs to the atmosphere...belongs to the way...belongs to all of me."

The atmosphere that pervades in the home is affected by its natural surroundings. The views and closeness to nature make one feel at home in the residency and the surrounding area and give one a feeling of well being. One thinks that a dwelling without a view would be somewhat depressing. The ability to follow seasonal changes throughout the year by looking out the window or by opening the door means very much. Everything that happens within and outside the home affects the atmosphere pervading in the home.

"Nature means a lot and that one can go outside and sit here, just exist."

Memories from the childhood home, where the older adult has one's roots, never disappear. They become a part of oneself and a part of the atmosphere in the current home. These memories are highly valued and carried by the older adult throughout life and provide a solid grounding in life.

"I think the childhood home never fully disappears; it has kind of remained."

A spiritual home, an invisible home with its foundation in church and ultimately in heaven, can become a part of the atmosphere pervading in a home. This spiritual home is nurtured by religious experiences in the home or in the church.

"The spiritual home is a home one never really has to leave...I experience a great safety in this that one like has a spiritual home too."

Thus, for the older adult home has a special atmosphere formed by one's personal values, surroundings and way of life. Closeness to nature and surrounding views outside the home affect and become a part of the atmosphere pervading in the home. A home can be seen as part of a more encompassing spiritual home. The atmosphere in the home gives the older adult a feeling of safety and brings peace to one's soul. When one is closely connected with one's inner self there is a feeling of being at home, a sense of integrity and wholeness.

### The home, a place the older adult might be forced to leave

The first main theme gives the image of home as an ideal place. However, concerns and worries about the future emerged in the interpretation of the text. There were things about home that were disconcerting and troubling. This included the fear of being forced to leave one's home, the day when there is no other way out, and the struggle not to think about it, the day the older adult does not want to think about.

#### The day when there is no other way out

The older adult is aware that home is a place that one might be forced to leave one day. It is something one has to do when the body has let one down, when affected by illness or when one has lost one's strength due to increased age. To leave one's home is nothing one will do voluntarily. One would have to be forced to leave, the time when there is no other way out. The older adult knows that such a time will come.

"What is closest to one's heart one does not want to loose. And one wants to keep it for as long as possible....One will not have a free will then, it is just a constraint."

"Well, of course one does not do it until one really has to, really has to do it...not me, at least."

The older adult did not question how or when or even if they would have to leave there home but assumed that they might be forced to leave. In the meantime they wanted to be there as long as possible and had no inclination or desire to think ahead and plan for that day.

#### The day the older adult does not want to think about

The older adult is aware that the home is something one might be forced to leave due to different reasons. However, one is determined to keep the home and live there as long as possible. When the day comes, when one is forced to leave one's home, this is a decision one wants to make by oneself. The anticipated day is something the older adult doesn't dare or would rather not think about, it feels too hard.

"The day when one has to, it will come. I think it will be hard...I can not imagine, I do not want to think about that day...It feels hard to think about it."

"Can not really imagine having to move...it will be a constraint. I don't dare to think about it."

The prospect of loosing ones home is so emotionally distressful that the older adult doesn't even dare to think about it or one thinks about it momentarily and deliberately chooses not to think about it further.

### The new understanding

In the new understanding the complexity of the older adult's experience of home emerged as depicted in Figure 1.

As seen above, this new understanding reflects the older adult's vacillation between thinking about their home as they are now experiencing it and fears and internal struggles around the day when they might be forced to leave their home. This sense of the future loss, when they might loose their home, was so strong and so threatening that the thought of it only emerged momentarily before it was consciously repressed. The older adult's thoughts vacillated between the well known present and all its comforts and the unknown future with all its questions and fears.

There were two ways in which the older adult thought that they might be forced to leave their home, one of which was to have to move into a residential facility for older adults. The older adult thought about how hard it would be to let go of things and see oneself move into that kind of environment and perceive it as a home. It was seen as an in-personal place, one where the older adult would be kept, and the freedom one had had in one's own home would be lost. Another possibility was if home health care providers entered or expanded their role in the home in such a way that it disrupted the older adults feeling of "being at home".

## Discussion

Some of the findings in this study lend empirical support to earlier philosophical analyses of home. For the older adults in this study, home was a place one could not imagine living without. It had become integral to living itself and an intimate part of the older adult's being. This merger of home with existence is not unlike Hilli's [44] description of home as ethos, which symbolizes the innermost room of a human being - the spirit. The empirical findings also are consistent with Dovey's [30] philosophical analysis of home as a mode of being. As noted earlier, Dovey [30] argued that home is an insider's unique experience, a mode of being that comes out of dwelling activities. This makes one wonder at what point in life the home moves from being a place to an actual mode of being. This also lends further support to Zingmark and Norberg's [35] call for further research on the experience of home and how it develops and changes over one's life time.

In the interviews, the older adults initially talked much about when and how they started to build their home with their spouses. However, as time passed by the circumstances in life changed and in the older adults' current experience of home the reference to others had changed. The relationship with family and friends were still important in life but the experience of home developed and became the older adults' stronghold and freedom which allowed them to decide on whom to keep in contact with and welcome and to whom they would open the door.

Other findings in this study illuminate a new understanding not previously covered in the literature where the home has generally been seen as a place of comfort not struggle [4,10,18,19,45,46]. The older adults in this study feared that the home might not continue to be a nourishing place and the central source of strength in daily life. Their thoughts vacillated between the well known from where they drew strength and the unknown which might drain their energy. There is a fear that home will become an unknown and an uncertain place. One might loose one's home, identity, integrity and way of living.

Hilli [44] argued that one can feel homeless in one's own home, when it has lost its special atmosphere. The older adults in this study saw this as a real possibility. They feared that their home could become so disrupted by health care providers that one might no longer feel one could be oneself. Actually, Hawkin and Stewart [24] have argued that in fact there is a tendency among health care providers to see the home as neutral territory, one where new relationships, occupational roles and functions can be incorporated rather easily, without problems. Furthermore, Steward [27] and Williams [11] have argued that the mere presence of health care providers in the home changes it from a private space to a public arena and may in turn disrupt the boundaries and the older adult's sense of home and well-being.

### Methodological considerations

The hermeneutical approach made it possible to capture, give expression to and make explicit an understanding [38] of the older adults' experience of home. However, this study has not produced knowledge that can be generalized to all older adults living in Sweden, although it can be reflected upon and considered in other settings where older adults live, such as residential facilities for older adults. Different themes might have been found if the older adults had been currently living in abusive situations. There was no indication of abuse in this group, however that remains unknown since no effort was made to directly probe for this.

As noted by von Post and Eriksson [37] and Gadamer [38], the origin of the text is not of primary importance but it is the text itself and the reader's dialogue with the text that is the focus of attention. The primary author, with the existential and professional pre-understandings [37] was familiar with what the older adults were saying and saw it as valid. The professional pre-understanding may also have prevented the primary author from seeing what was hidden behind the words, because what was heard seemed obvious and something over which there seemed to be no need to reflect further. In qualitative research, content validity is one of the standards to validate that the chosen method investigates the intended content. The validity of the themes in this study was strengthened since both co-authors acted as co-judges examining the themes separately [42].

In the interviews, older adults initially had difficulty talking about home, since it generally had been taken for granted. According to Dovey [31], the experience of home is largely unselfconscious and unrecognized until threatened. One question asked the older adults was to think about the possibility of loosing their home. The question, unintentionally, may have felt like a threat allowing the older adult to recognize and more clearly articulate the experience of home and their fears of loosing it. Also, the active interactional dialogue that took place when the husband and wife were interviewed together would suggest that group interviews might help stimulate recall, deepen the dialogue and lead to a fuller and more complex text for interpretation.

## Conclusions

This study provides empirical support for how precious and close to the heart experience of home can be for the older adult, being intimately linked with one's identity, integrity and a way of living as well as a hidden struggle between the comfort of the well-known and fears of the unknown, including the underlying threat of loosing one's home. The vacillation and the ongoing tension between the well known, which gave them strength, and the unknown, which caused them distress.

These findings could be important for nurses and other health care providers. Health care providers might want to consider setting aside time for a dialogue with the older adult to talk about his or her experiences of home in order to give as much attention to minimizing intrusion as to maximizing care. Additionally, researchers need to explore more fully older adults' experiences with health care providers in their homes. What if any impact does this experience have on the older adult's sense of "being at home" and their health and overall well-being.

## Competing interests

The authors declare that they have no competing interests.

## Authors' contributions

The first author (CG) was the primary author and originator of the study and designed the study, collected and analyzed the data and prepared the manuscript for submission. The second author (D S-B) and third author (I vP) participated in a supportive role in all phases of the study. All authors contributed to the manuscript and have read and approved the final manuscript.

## Pre-publication history

The pre-publication history for this paper can be accessed here:

http://www.biomedcentral.com/1471-2318/11/10/prepub
